# Hemicranial Cough-Induced Headache as a First Symptom of a Carotid-Cavernous Fistula-Case Report

**DOI:** 10.3390/medicina56040194

**Published:** 2020-04-23

**Authors:** Svetlana Simić, Ljiljana Radmilo, José R. Villar, Aleksandar Kopitović, Dragan Simić

**Affiliations:** 1 Department of Neurology, Faculty of Medicine, University of Novi Sad, Hajduk Veljkova 1–9, 21000 Novi Sad, Serbia; aleksandar.kopitovic@mf.uns.ac.rs; 2 Department of Neurology, General Hospital “Dr Radivoj Simonović”, 25000 Sombor, Serbia; ljiljanardml@gmail.com; 3 Faculty of Geology, University of Oviedo, Campus de Llamaquique, s/n, 33005 Oviedo, Spain; villarjose@uniovi.es; 4 Faculty of Technical Sciences, University of Novi Sad, TrgDositejaObradovića 6, 21000 Novi Sad, Serbia; dsimic@uns.ac.rs

**Keywords:** headache, internal carotid artery, carotid cavernous sinus fistula, spontaneous resolution

## Abstract

*Background and objectives:* Spontaneous carotid-cavernous fistulas (CCFs) are rare, and they may be caused by an aneurysm rupture. *Materials and Methods:* A case of a man hospitalized for high-intensity hemicranial headache with sudden cough onset as part of an upper respiratory tract infection is presented. The pain was of a pulsating character, localized on the right, behind the eye, followed by nausea and vomiting. Neurological finding registered a wider rima oculi to the right and slight neck rigidity. Laboratory findings detected a mild leukocytosis with neutrophil predominance, while cytobiochemical findings of CSF and a computerized tomography (CT) scan of the endocranium were normal. *Results:* Magnetic resonance imaging (MRI) angiography indicated the presence of a carotid cavernous fistula with a pseudoaneurysm to the right. Digital subtraction angiography (DSA) was performed to confirm the existence of the fistula. The planned artificial embolization was not performed because a complete occlusion of the fistula occurred during angiographic examination. Patient was discharged without subjective complaints and with normal neurological findings. *Conclusions:* Hemicranial cough-induced headache may be the first sign of carotid cavernous fistula, which was resolved by a spontaneous thrombosis in preparation for artificial embolization.

## 1. Introduction

Carotid-cavernous fistula (CCF) is a pathological communication of the carotid arteries or their branches with the cavernous sinus [1,2,3,4,5]. In relation to cause, they are divided into traumatic, spontaneous, and iatrogenic. They are mostly traumatic (in 70% of cases) [1]. Spontaneous events are much rarer and may be caused by ruptured aneurysm, atherosclerosis, or blood vessel inflammation [6]. In terms of the flow velocity, they are divided into low and fast flow fistulas [3]. Direct fistulas are those in which there is a direct communication between the internal carotid artery and the cavernous sinus, and the indirect are the ones that occur between the meningeal branches and the cavernous sinus1. High-pressure arterial blood is mixed with the venous blood of the cavernous sinus, resulting in retrograde flow through the veins draining into the cavernous sinus [2,6]. Venous drainage of CCF can be anterior via the ophthalmic veins or posterior via sinus petrosus. Drainage is usually mixed [1]. The type of drainage conditions the clinical picture [3,5,6]. If there is an anterior drainage, the clinical picture is mainly accompanied by ocular symptomatology ranging from pulsating exophthalmos, conjunctival chemosis, thickening and tortuosity of the scleral vessels, signs of elevated intraocular pressure, nystagmus, impaired mobility of the eye to ophthalmoplegia, diplopia, and the decrease in visual acuity, while posterior drainage is characterized by an impression of a humming noise in the head. Both types of drainage can cause headache [6]. These symptoms are usually ipsilateral to the fistula [2]. 

## 2. Case Report

A 32-year-old man was hospitalized because of an intensive hemicranial headache, caused by coughing. Three weeks before the admission, a dry cough appeared, and after a few days, a fever, which lasted for 4–5 days. His pulmonologist prescribed an antibiotic due to the upper respiratory tract infection. A few days after the administration of the antibiotic, febrility disappeared, but the dry cough persisted. Two weeks before admission, a cough was accompanied by a headache, which persisted and increased with the cough. The pulsating pain with nausea and vomiting was localized on the right, behind the eye, with no other accompanying symptoms. Since the headache persisted, he was referred to a neurologist, who verified a normal neurological findings. Laboratory findings detected mild leukocytosis, with neutrophil predominance, without other inflammation parameters. Given the prolonged hemicranial headache, although the neurological finding was normal, a computerized tomography (CT) scan of the endocranium was made, and the scan was described as normal. A lumbar puncture was then performed to obtain clear liquor, and, again, the cytobiochemical finding was normal. With symptomatic therapy, the patient was referred to the Headache Cabinet for suspected migraine without aura. The next day the headache began to worsen in a standing position, and the neck pain occurred as well, probably due to the associated dural-puncture headache (PDPH). The patient was examined in the Headache Cabinet and immediately hospitalized due to prolonged hemicranial headache that did not meet any primary headache diagnostic criteria and did not have, according to the diagnostic test performed, any etiologic cause [7]. The pattern of this headache was a “red flag” and indicated the need to determine the etiological cause of the headache [8]. 

**Figure 1:**
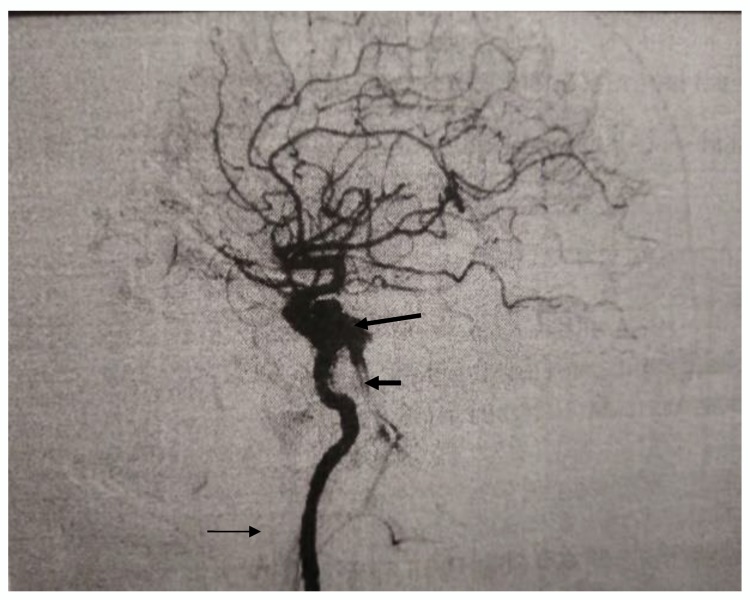
Digital Subtraction Angiography (DSA) finding before the spontaneous occlusion demonstrating carotid-cavernous fistula (long arrow) with enlarged vein (short arrow). Right internal carotid artery (thin arrow).

On admission, the patient said he has never had a headache or any other illness, injury, or surgery. He recreationally played football. His neurological finding, on admission, showed a discreetly broader rima oculi to the right and tightening of the neck during anteflexion, while the other neurological findings were completely normal. An emergency neuroradiological Magnetic Resonance Imaging (MRI) of the endocranium with arteriography and venography was performed, and it indicated the saccular formation of approximately 7.5 × 5.5 mm, with the lateral contour of the right internal carotid artery in the region of the cavernous sinus insuspected communication with the C4-5 segment of the right internal carotid artery. It also indicated the dilation of the ophthalmic vein and the veins along the wing of the sphenoid bone, the cavernous sinus itself, as well as the sinus by the clivus. The described finding spoke in favor of CCF with pseudoaneurysm/aneurysm. After these findings, the patient was immediately transferred to the Clinic for Neurosurgery, and the Digital Subtraction Angiography (DSA) (Figure 1) performed there confirmed the presence of CCF to the right, with an aneurysm of 7 mm in diameter, from which the fistula into the cavernous sinus probably arose. Since the fistula opening was small, there was no technical possibility to close it and the patient was transferred to another institution for artificial embolization with stent assistance. Angiography of the blood vessels of the brain was performed there, and it indicated a subocclusion of the previously angiographically proven carotid-cavernous fistula. 

**Figure 2:**
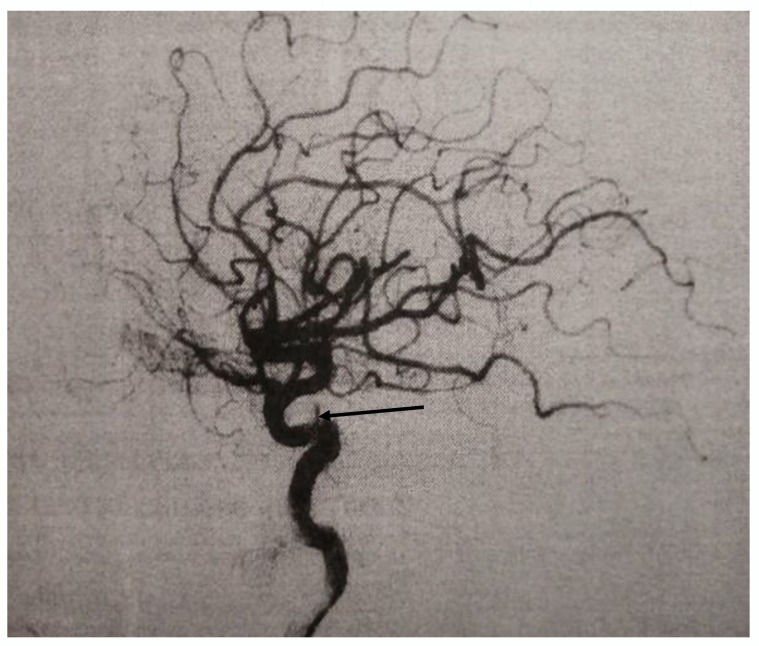
DSA finding after the spontaneous occlusion, arrow indicate cavernous segment of the internal carotid artery.

During the angiographic examination, complete occlusion of the fistula occurred (Figure 2), and the planned procedure was abandoned. Patient was discharged with normal neurological findings, with recommendation for occasional compression of the right carotid artery. After four months, a control DSA was made and it did not register CCF but a slight enlargement (up to 2 mm) on the intracranial segment of the right internal carotid artery, which might correspond to the site of the earlier fistulose duct. 

**Figure 3:**
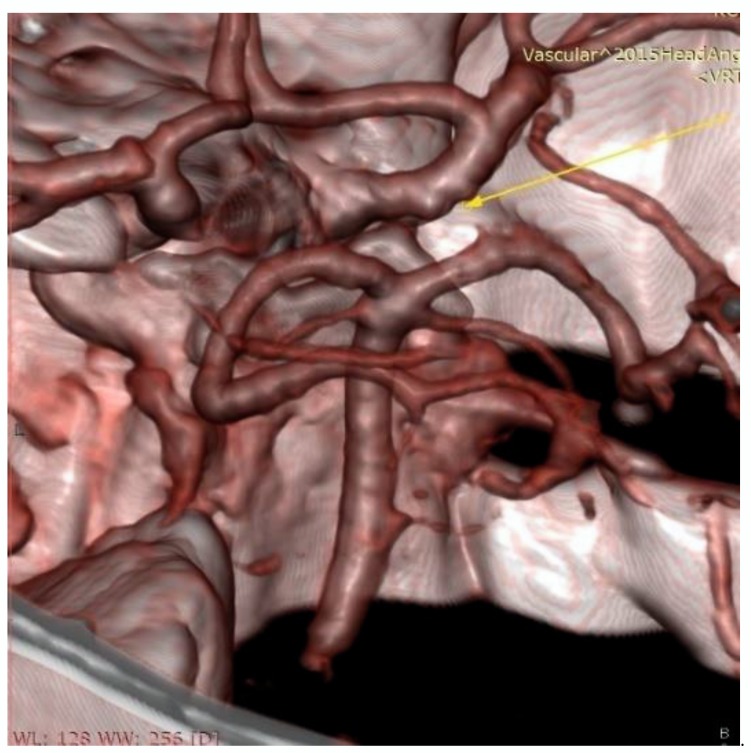
Control Magnetic Resonance Angiography (MRA), arrow indicate the slight enlargement on the intracranial cavernous segment of the right internal carotid artery.

Furthermore, the patient is regularly monitored by a neuroradiologist. MR brain angiography (Figure 3) was performed and the findings on the blood vessels remained unchanged in relation to the previously performed DSA. This review was approved by the Ethics Committee of the Clinical Centre of Vojvodinaon 27 December 2019 (No.00-1168). Informed written consent was obtained from the patient.

## 3. Discussion

Differential diagnosis of hemicranial pain involves many primary and secondary headaches and painful cranial neuropathies. As unilateral pain may be a characteristic of primary headaches, secondary headaches and painful cranial neuropathies, whose clinical presentations often overlap, differential diagnostic dilemmas are often encountered in clinical practice in determining the cause of a hemicranial headache [8]. Since our patient had a cough-induced headache, one could think of a differential diagnosis leading to the primary cough headache (Table 1), but the pattern of the headache and the new sudden headache lead this diagnosis to be dismissed. 

The reference diagnosis of a migraine without aura was also rejected on anamnestic data themselves, because the criteria given in the International Classification of Headaches (ICHD-3) [7] were not met. Sudden onset hemicranial primary headache are chronic headache with episodic headache attacks. Clinically our patient had first onset of severe headache, with a duration of three weeks, excludes migraine, and without other accompanying migraine and cranial autonomic symptoms, which exclude trigeminal autonomic cephalalgias such as cluster headache, paroxysmal hemicranias, short-lasting unilateral neuralgiform headache attacks, short-lasting unilateral neuralgiform headache attacks with cranial autonomic symptoms, hemicrania continua. Attention was focused on identifying the underlying cause for the secondary headache in our patient. Research to date shows that in 40% of cases, cough-induced headache was secondary. In most cases, this type of headache is primarily caused by Arnold–Chiari malformation type I and then by other causes (cerebral aneurysms, tumors of the middle and posterior cranial fossa, midbrain cyst, subdural hematoma, reversible cerebral vasoconstriction syndrome, carotid and vertebrobasilar disease, etc.) [7]. Severe sudden onset secondary headaches present neurological emergences when there is a need to think about subarachnoid hemorrhage, all reasons for raised intracranial pressure, and cerebral infection. Neurological examination, normal CT scan, and normal CSF exclude many of these conditions. For more detailed neuroimaging we performed MRI of the endocranium with arteriography, and because of previous infection we doubted the cerebral venous thrombosis and performed MRI of the endocranium with venography. Since the diagnostic procedures determined the existence of CCF. Using the criteria given in ICHD-3, we diagnosed the patient with headache attributed to dural arteriovenous fistula (DAVF) (Table 2). 

Barrow et al.areclassified CCF into 4 types [9].Type A are fistulas between the internal carotid artery or its branches with the cavernous sinus characterized by rapid and high flow, and are divided into two subtypes: A1, which are most traumatic in origin, and A2, which are the result of rupture of the internal carotid artery aneurysm in the cavernous sinus. Type B are the dural shunts of the meningeal branches of the internal carotid artery with the cavernous sinus. Type C is a dural shunt of the meningeal branches of the external carotid artery with a cavernous sinus, and type D is a combination of types B and C. Types B, C, and D are characterized by low flow [1,3,5,6,10]. Our patient, had CCF type A2 caused by an aneurysm rupture that most likely occurred with a cough. It has been reported in the literature that rupture of an intercavernous aneurysm may be accompanied by epistaxis and subarachnoid hemorrhage [11]. What is unusual and differentiates our case from other published case reports with CCF type A2 is that, apart from the headache, there was no other symptoms that would indicate a fistula. In addition to hemicranial headache, with ipsilateral retroorbital pulsatile pain and discretely wider rima oculi to the right, no other ocular symptoms were present, and there was no pulsating tinnitus, although the findings of MR angiography and venography of the endocranium described dilation of the ophthalmic vein and the veins along the wing of the sphenoid bone, the cavernous sinus itself, and the sinus by the clivus. This confirms that hemicranial headache is itself a “red flag,” requiring detailed diagnostics to rule out a secondary headache and to etiologically direct treatment [8]. The absence of other accompanying clinical manifestations in our patient can be explained by the fact that headache is usually the first clinical manifestation, followed by other symptoms. An unusual clinical presentation of unilateral CCF in the form of frontal localization headache and bilateral ocular manifestations was recently reported by Demartini et al. [2]. Namely, it is known and expected that a bilateral clinical manifestation accompanies bilateral CCF, not unilateral [6]. The first case, in the literature, of post-traumatic CCF, accompanied by contralateral ocular manifestations and a headache, has recently been reported. This clinical manifestation is thought to be caused by the contralateral flow of the fistula across the branches of the external carotid artery [4]. 

In their work, Celik et al. emphasize that the presence of unilateral ocular manifestations occurring as part of the clinical picture of CCF is often misdiagnosed as Graves’ ophthalmopathy or inflammatory conjunctivitis [12]. Since this leads to a delay in the diagnosis of CCF, the authors of this particular study recommend that additional test, particularly DSA, should be performed in all cases of unilateral ocular manifestations that were not alleviated by standard treatments [12]. Timely diagnosis and adequate treatment play a key role in the prognosis, as they can prevent the complications that CCF can cause, the most serious of which are blindness, intracranial hemorrhage [6], and venous infarctions [3]. Given that endovascular therapy has proven to be the most successful therapy with very few complications [3,13], our patient was immediately referred to a neurosurgeon for artificial embolization, which, due to spontaneous thrombosis of the fistula, was not performed. Spontaneous thrombosis of type A fistula due to direct communication, and rapid flow happens very rarely [5]. However, it has also been described in multiple cases [5,10,13]. Uchino A. and associates provide two potential explanations for why their patient experienced spontaneous CCF thrombosis after a failed balloon-occluded transarterial chemoembolization. Specifically, in their patient, the inflated balloon was, repeatedly, inserted in the fistula opening leading to its occasional occlusion and decreased blood flow and stagnation, which may have played a role in the formation of the thrombus. This could explain the recommendation that in direct CCF the common carotid artery should be compressed, since the compression leads to arterial hypotension and venous hypertension, which further causes a transient decrease in pressure gradient across the shunt, which promotes thrombosis. Their second explanation is that the injection of iodine contrast agent played an important role [10]. A different explanation for the occurrence of spontaneous thrombosis of CCF, is provided by Naragum et al. Specifically, they believe that the healing of arterial injury caused by catheter manipulation during angiographic processing causes spontaneous thrombosis [5].

## 4. Conclusions

Hemicranial cough-induced headache may be the first sign of CCF, and in such cases, a fistula should be suspected, and detailed diagnostic tests should be performed in order to diagnose and prevent serious complications of CCF rupture. In direct CCF, a spontaneous thrombosis can occur due to angiographic processing.

## Figures and Tables

**Table 1:** Diagnostic criteria for primary cough headache.

ICHD-3 Diagnostic Criteria for Primary Cough Headache	
**A**.	At least two headache episodes fulfilling criteria B–D
**B.**	Brought on by and occurring only in association with coughing, straining and/or other Valsalva maneuvre
**C.**	Sudden onset
**D.**	Lasting between one second and two hours
**E.**	Not better accounted for by another ICHD-3 diagnosis

A. The minimum number of attacks required for diagnosis; B. Potential triggers for attacks; C. Onset of the attack; D. Attack duration; E. Consideration of other possible diagnoses.

**Table 2:** Headache attributed to dural arteriovenous fistula (DAVF).

ICHD-3 Diagnostic Criteria for Headache Attributed to Dural Arteriovenous Fistula			
A.	Any new headache fulfilling criterion C		
B.	A dural arteriovenous fistula (DAVF) has been diagnosed		
C.	Evidence of causation demonstrated by at least two of the following:		
	1.	Headache has developed in close temporal relation to other symptoms and/or clinical signs of DAVF, or has led to the diagnosis of DAVF	
	2.	Either or both of the following:	
		(a)	Headache has significantly worsenedin parallel with other symptoms or clinical or radiological signs of growth of the DAVF
		(b)	Headache has significantly improved or resolved after effective treatment of the DAVF
	3.	At least one of the following:	
		(a)	Headache is accompanied by pulsatile tinnitus
		(b)	Headache is accompanied by ophthalmoplegia
		(c)	Headache is both progressive and worse in the morning and/or during coughing and/or bending over
	4.	Headache is localized to the site of the DAVF	
D.	Not better accounted for by another ICHD-3 diagnosis		
